# Adult intussusception due to ileal polyp - A case report

**DOI:** 10.1016/j.ijscr.2022.107554

**Published:** 2022-08-26

**Authors:** Tahmina Hakim

**Affiliations:** Department: General Surgery, Campbelltown Hospital, 6 Quail Place, Ingleburn, NSW 2565, Australia

**Keywords:** Intussusception, Bowel obstruction, Intestinal polyps

## Abstract

**Introduction:**

Intussusception can be potential cause of bowel obstruction. Varying in clinical aetiologies, intestinal polyps are the most common cause where the possibilities of malignancy increase from proximal to distal colon. Laparoscopic reduction can be considered to prevent recurrence and complications of intussusception.

**Case presentation:**

A case report has been discussed where small bowel polyp caused intussusception which was diagnosed with Magnetic Resonance Enterography and managed with laparoscopy and bowel resection with anastomosis. 27 years old lady presented with colicky abdominal pain with no tenderness. Her initial CT abdomen showed ileo-ileal intussusception with possible soft tissue lesion. Her Magnetic Resonance Enterography (MRE) demonstrated tubular lipomatous polyp causing intussusception. She underwent elective diagnostic laparoscopy with bowel resection and anastomosis.

**Clinical discussion:**

Intussusception is one of the common causes of intestinal obstruction which can be caused by intestinal polyp. With variable clinical features, intussusception can be diagnosed with CT scan. Laparoscopic procedure can be performed in case of symptomatic intestinal obstruction caused by intussusception.

**Conclusion:**

CT abdomen can also be considered to diagnose intussusception where radiological feature of lead point can be diagnostic. Bowel resection is required in most of the cases to reduce complications of intussusception.

## Highlights

• Intestinal lipomas are the most common cause of intussusception in adults.
• Identification of lead point on CT/MRI is a radiological indicator in diagnosing intussusception.
• As intussusception reduction has the potential risk of bowel perforation, bowel resection should be accomplished in most cases.

## 1 Introduction

Intussusception can be defined as telescoping of a bowel segment into distal segment. The clinical features of intussusception are variable in nature varying from abdominal pain, nausea, vomiting, constipation depending on location of aetiologies. One of the most common causes of small bowel intussusception is benign polyps which can be diagnosed by CT abdomen with contrast. To rule out possibilities of malignancies and prevent recurrence with complications, laparoscopic reduction with or without laparotomy can be considered.

## 2 Case presentation

**Image 1:**
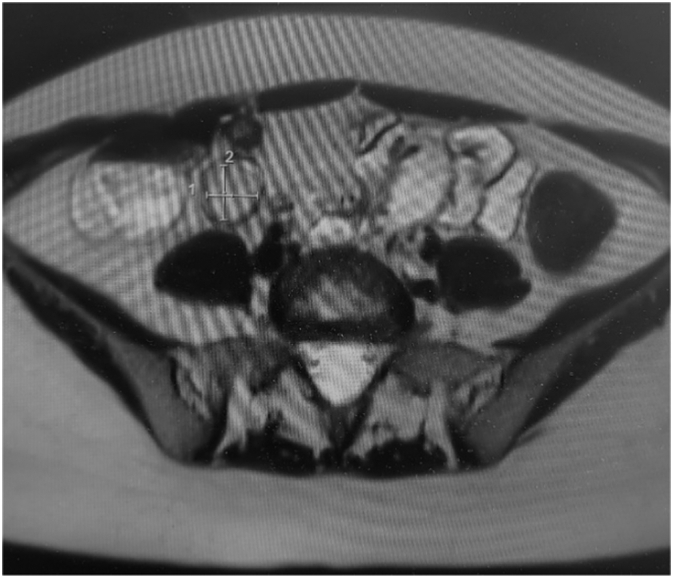
Magnetic Resonance Imaging Enterogram showing heterogenous T2 iso hyperinterse lesion within distal ileum.

**Image 2:**
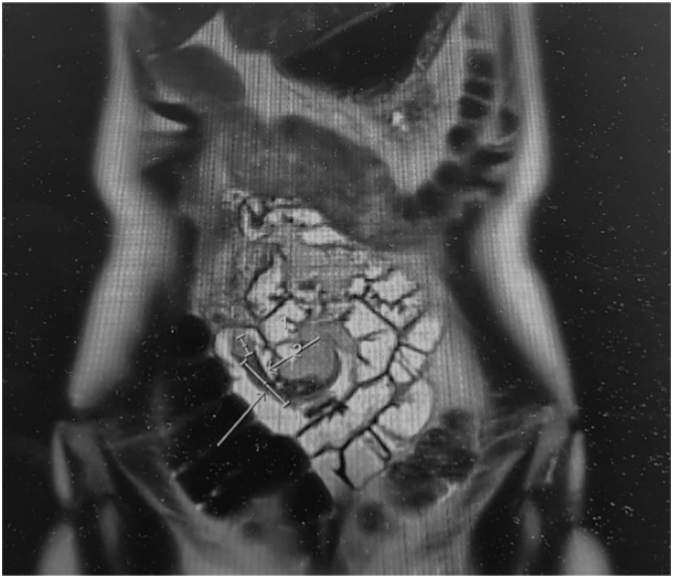
Elongated tubular structure likely representing ileo-ileal istussusception.

**Image 3:**
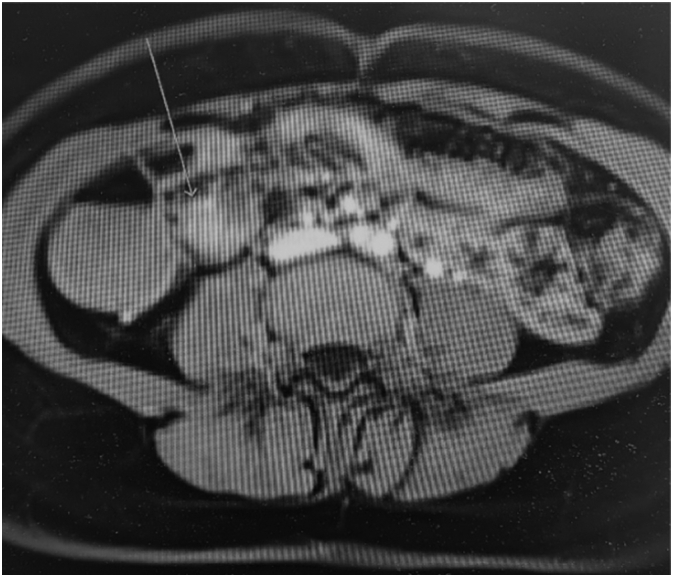
Double lumen appearance of ileo-ileal intussusception demonstrating internal fat.

**Image 4:**
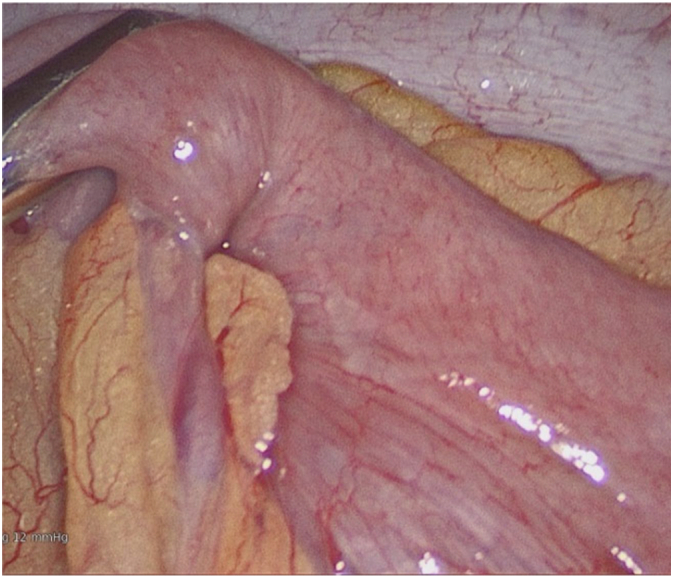
Distal ileal tubular structure.

**Image 5:**
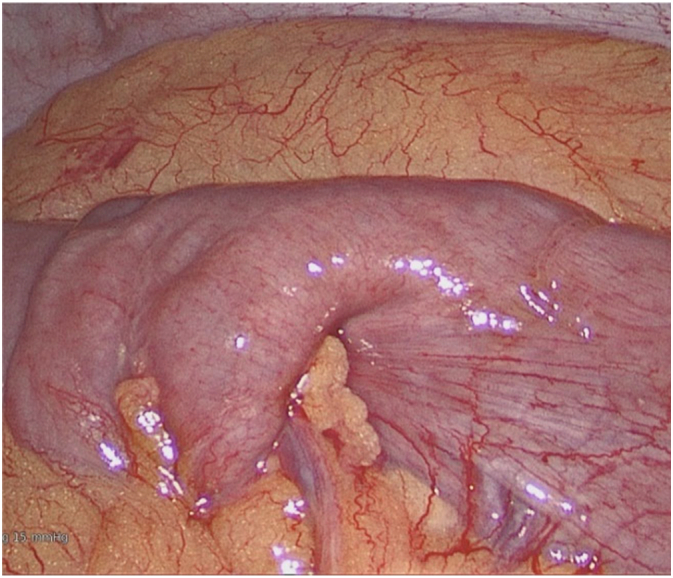
Ileo-ileal intussusception.

**Image 6:**
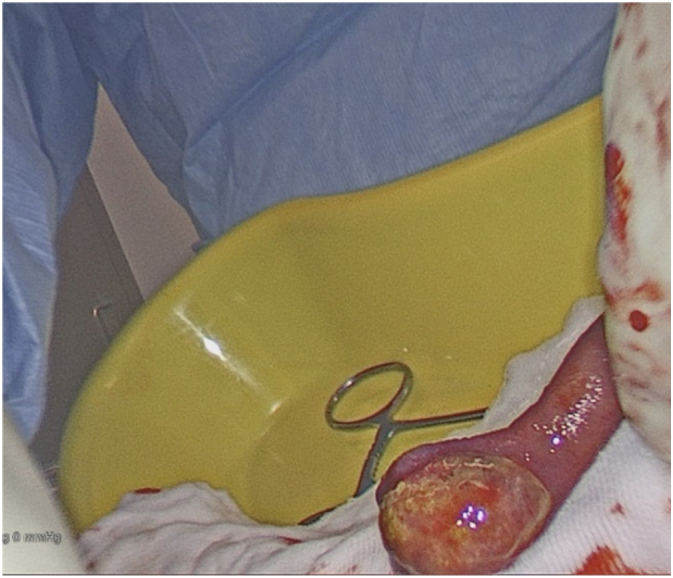
Intesinal fibroid lipoma.

27 years old lady presented to ED with 2 weeks of intermittent, colicky abdominal pain which resolved spontaneously in 20-30 min. Her initial CT showed ileo-ileal intussusception and possible soft tissue lesion. She did not have any significant medical history. On examination, her abdomen was soft and non-tender. Her WBC was 11.2 with normal CRP. Her repeat CT demonstrated elongated pedunculated fat-containing filling defect in the proximal ileum, likely lipomatous polyp, ileo-ileal intussusception appears to have resolved. Her pain was resolved on same day and she was discharged with outpatient MRI enterogram which showed within the distal ileum, there is heterogenous hyperintense lesion measuring 18 × 17 × 10 mm (Image 1). Associated with this, there is an elongated tubular structure measuring 31 mm appearing to have double lumen appearance (Image 2, Image 3). There is a fat based lesion within the distal ileum (Image 4, Image 5). The elongated attached tubular structure is likely to represent a focal area of ileo-ileal intussusception with the fat based lesion acting as a lead point rather than elongated stalk (Image 6). It is most likely to represent a lipomatous polyp, however it has some heterogenous enhancement which is atypical, possibly due to intussusception. After 2 weeks, she was booked for diagnostic laparoscopy on elective list which demonstrated mid ileal intra-luminal 2 × 2 cm lipoma causing intussusception (Image 6), rest of the small bowel was normal. Small bowel resection was performed including lipoma with side-to-side functional and end-to-end small bowel anastomosis. The umbilical port was extended to exteriorize small bowel specimen. Post-operatively she improved well with gradual upgrade of diet. Her histopathology revealed sub-mucosal lipoma with mucosal ulceration and acute on chronic inflammation.

## 3 Discussion

Intussusception can be described as a condition where one gastrointestinal segment is telescoped into nearby distal segment. Intussusception of small bowel usually instigated by benign aetiologies and one of the rare causes is inflammatory fibroid polyp (IFP) which is most commonly found in ileum. Varying in clinical features, presentation of IFPs can be asymptomatic, pain in abdomen and bowel obstruction in rare condition. With ambiguous pathogenesis, eosinophilia was found as potential reason causing IFPs. Usually IFPs are pedunculated or sessile lesion evolving from submucosa of bowel lumen with mucosal ulceration, measuring usually 2-5 cm diameter [1].

Predominantly the aetiologies of small bowel intussusception are of benign processes and large bowel intussusception is of malignant processes. The possibility of malignancy increases from proximal to distal. Benign aetiology like lipomas is the most common cause of intussusception in adult [2].

Due to probability of malignancy and pathological abnormalities, adult intussusceptions are managed by surgical procedures like laparotomy and laparoscopy. There is existence of some disagreement on whether intussusception reduction might minimise the magnitude of resection of bowels. However, improper reduction may increase the risk of perforation of bowels, complications of anastomosis, propagation of malignancy. Therefore, to avoid all these potential complications, bowel resection has been accomplished in most of the cases [3].

CT is an important tool for diagnosing intussusception and assessing complications determining lesion's extent and location. Identification of a lead point on CT is a consistent radiological indicator for diagnosing intussusception [4].

## Provenance and peer review

Not commissioned, externally peer-reviewed.

The work has been reported in line with the SCARE 2020 criteria [5].

## Funding

No funding required.

## Ethical approval

No approval required.

## Consent

Verbal consent obtained.

## Author contribution

Tahmina Hakim is the sole author for the case report.

## Registration of research studies

Not applicable.

## Guarantor

TAHMINA HAKIM.

## Declaration of competing interest

No conflict of interest.
